# Identity-by-descent-based heritability analysis in the Northern Finland Birth Cohort

**DOI:** 10.1007/s00439-012-1230-y

**Published:** 2012-09-29

**Authors:** Sharon R. Browning, Brian L. Browning

**Affiliations:** 1 Department of Biostatistics, University of Washington, Seattle, WA USA; 2 Division of Medical Genetics, Department of Medicine, University of Washington, Seattle, WA USA

## Abstract

For most complex traits, only a small proportion of heritability is explained by statistically significant associations from genome-wide association studies (GWAS). In order to determine how much heritability can potentially be explained through larger GWAS, several different approaches for estimating total narrow-sense heritability from GWAS data have recently been proposed. These methods include variance components with relatedness estimates from allele-sharing, variance components with relatedness estimates from identity-by-descent (IBD) segments, and regression of phenotypic correlation on relatedness estimates from IBD segments. These methods have not previously been compared on real or simulated data. We analyze the narrow-sense heritability of nine metabolic traits in the Northern Finland Birth Cohort (NFBC) using these methods. We find substantial estimated heritability for several traits, including LDL cholesterol (54 % heritability), HDL cholesterol (46 % heritability), and fasting glucose levels (39 % heritability). Estimates of heritability from the regression-based approach are much lower than variance component estimates in these data, which may be due to the presence of strong population structure. We also investigate the accuracy of the competing approaches using simulated phenotypes based on genotype data from the NFBC. The simulation results substantiate the downward bias of the regression-based approach in the presence of population structure.

**Electronic supplementary material:**

The online version of this article (doi:10.1007/s00439-012-1230-y) contains supplementary material, which is available to authorized users.

## Introduction

Genome-wide association studies (GWAS) have been successful in finding a large number of variants associated with common diseases. Over 1,000 such associations have been documented to date (http://www.genome.gov/gwastudies/) (Hindorff et al. 2009). However, the associated variants have mostly been of small effect, and the cumulative proportion of heritability explained for each disease remains small for most diseases (Manolio et al. 2009). Several explanations have been proposed for this missing heritability, including the effects of rare variants and other variants not well-tagged by SNP arrays, the existence of large numbers of causal loci of very small effect, the contribution of gene–gene and gene–environment interactions, and insufficient adjustment for shared environment between related individuals (Manolio et al. 2009).

Heritability can be measured in a broad-sense, which measures the full contribution of all genes, or it can be measured in a narrow-sense, which measures only additive effects (Visscher et al. 2008). Studies of missing heritability focus on narrow-sense heritability because it is much easier to measure. Effect sizes of individual causal variants can be estimated from genetic association studies, and these effects can be summed over all of the known causal variants to obtain an estimate of the narrow-sense heritability that has been explained by these variants. However, one cannot determine how much the set of known variants contributes to broad-sense heritability because there may be many complex interactions between causal variants in different genes that have effects that are too small to be detected (Zuk et al. 2012). Estimates of heritability are usually derived from twin studies and other studies of close relatives, and such estimates include not only the additive portion of heritability, but also some effects of gene–gene and gene–environment interactions (Zuk et al. 2012; Falconer and Mackay 1996). Zuk et al. (2012) have shown that narrow-sense heritability could be substantially smaller than estimates of heritability from studies of related individuals, and consequently GWAS may explain a higher proportion of narrow-sense heritability than previously thought. Our focus in this study is on estimating narrow-sense heritability.

Zuk et al. (2012) propose a method for obtaining an unbiased estimate of narrow-sense heritability. Their approach is to use a population sample, and regress phenotypic similarity on estimates of relatedness derived from detected segments of identity by descent (IBD). The use of a population sample rather than close relatives is key because this greatly reduces the incorporation of genetic interaction effects and shared environment effects into the heritability estimates (Zuk et al. 2012). The use of IBD segments in the estimation of relatedness is also very important, because IBD segments can incorporate the effects of any rare variants lying within the IBD segments (Zuk et al. 2012). In contrast, Yang et al. (2010) estimate relatedness using a method of moments estimator based on allele-sharing. Yang et al.’s (2010) estimate incorporates effects of the genotyped SNPs and other variants in strong linkage disequilibrium (LD) with those SNPs, but does not include the effects of most rare variants, which are in low LD with SNPs on genotyping arrays.

Heritability estimation using IBD segments works best in founder populations, because the more IBD that is present in the sample, the more precise is the estimate of heritability (i.e., the lower the standard error). In this article, we conduct genome-wide heritability analyses of the Northern Finland Birth Cohort (NFBC) 1966 GWAS data. Standard GWAS analyses of these data have been published previously, and multiple genome-wide significant associations were found (Sabatti et al. 2009). We compare several different approaches to estimating the heritability from the GWAS data.

First, we use the method of Zuk et al. (2012) in which products of normalized trait values are regressed against relatedness values obtained from detected IBD segments. The estimated heritability is obtained from the slope of the regression line (Zuk et al. 2012).

Second, we use the method of Yang et al. (2010) as implemented in the Genome-wide Complex Trait Analysis (GCTA) software (Yang et al. 2011). For this method, relatedness is estimated using allele-sharing. Heritability is estimated by fitting the variance component model.

1 $$ y = X\beta + g + \varepsilon \quad {\text{with}}\quad {\text{Var}}\left( g \right) = A\sigma_{g}^{2} {\text{ and Var}}\left( \varepsilon \right) = I\sigma_{\varepsilon }^{2} $$

where *y* is the vector of trait values, *X* is a matrix of covariates with effects given by the vector β, *g* is the sum of genetic effects from all autosomal loci, and ε is a vector of residual error, including environmental effects, *A* is the matrix of relatedness values which has correlations between allele dosage for pairs of individuals and estimated inbreeding coefficients on the diagonal, $\sigma_{g}^{2}$ is the genetic variance of the trait, *I* is the identity matrix, and $\sigma_{\varepsilon }^{2}$ is the residual or environmental variance. The heritability is ${{\sigma_{g}^{2} } \mathord{\left/ {\vphantom {{\sigma_{g}^{2} } {\left( {\sigma_{g}^{2} + \sigma_{\varepsilon }^{2} } \right)}}} \right. \kern-\nulldelimiterspace} {\left( {\sigma_{g}^{2} + \sigma_{\varepsilon }^{2} } \right)}}$.

Third, we estimate relatedness using detected IBD segments and incorporate this estimate in the variance component model of Yang et al. (2010). Our IBD-segment-based analyses with GCTA are similar to the work of Price et al. (2011) which used IBD-segment-based estimates of relatedness in a variance component approach to estimating heritability, although Price et al. focused on closely related pairs of individuals.

## Subjects and methods

### North Finland Birth Cohort

The NFBC GWAS data consist of 5,402 individuals from Northern Finland, born in 1966, with metabolic trait measurements and genotypes on 320,981 autosomal SNPs and 9,581 X chromosome SNPs from an Illumina Infinium SNP array. All individuals were of the same age (31-years old) at the time of the measurements. Covariate information includes sex, whether the individuals were taking medication for diabetes, whether they were taking oral contraceptives, whether they were pregnant, their fasting status, and whether their weight was self-measured. We excluded individuals who were pregnant (199 individuals) or taking diabetes medication (27 individuals). We estimated heritability for the nine metabolic traits measured in these data: body mass index (BMI), high-density lipoprotein (HDL) cholesterol, low-density lipoprotein (LDL) cholesterol, systolic blood pressure, diastolic blood pressure, glucose, insulin, triglycerides, and C-reactive protein (CRP). For analysis of BMI we excluded individuals who had self-measured weight (170 individuals). For triglycerides, insulin, glucose, HDL, and LDL, we excluded individuals who were not fasting at the time of measurement (228 individuals). As in Sabatti et al. (2009), CRP values were log-transformed after changing values of 0 to 0.002 (half the detection threshold), and BMI, triglyceride, insulin and glucose values were also log-transformed, because of the skewness of these phenotypic distributions. We used linear regression to adjust all traits for sex and oral contraceptive status. After this adjustment, we truncated all trait values that were more than 4 standard deviations from the mean to 4 standard deviations from the mean to reduce the impact of outliers.

### Estimation of relatedness using IBD segments

We detected IBD segments between pairs of individuals using a pre-release version of BEAGLE Refined IBD. This IBD detection method will be described elsewhere. In brief, it has similar computational performance to BEAGLE fastIBD (Browning and Browning 2011a), while having improved power and error rates. BEAGLE Refined IBD outputs IBD tracts for pairs of individuals; IBD is assumed to have 0–1 status (i.e., the possibility of bilineal relatedness, as in full-siblings, is not considered). The choice of parameter values for this method is described in Section 2 of the Appendix (Electronic supplementary material). For each pair of individuals we calculated the proportion of the genome shared IBD. 570 pairs of individuals shared IBD segments totaling over 750 cM in length. In an outbred population, we would expect to see this amount of IBD sharing in first-cousins or closer relatives; however, in an inbred population such as this one, individuals are related through many common ancestors, each of which can contribute some IBD. We identified one individual from each pair sharing over 750 cM of detected IBD segments, for a total 503 individuals (some of these individuals have more than one close relative in the data set). These 503 individuals were removed from all further analyses. The purpose of removing close relatives is to avoid confounding with shared environment and to reduce the influence of interactive effects (Visscher and Yang 2010; Zuk et al. 2012).

We turn now to the use of detected IBD segments to estimated relatedness. The relatedness value for a pair of individuals is twice their realized kinship coefficient. That is, the relatedness value is twice the probability that a random allele taken from each individual at a random location in the genome will be identical by descent. There are different ways to estimate this quantity. GCTA estimates the relatedness value based on normalized rates of allele-sharing (see below), while for the IBD-segment-based approaches we estimate it based on the quantity of IBD detected for the pair of individuals.

**Fig. 1:**
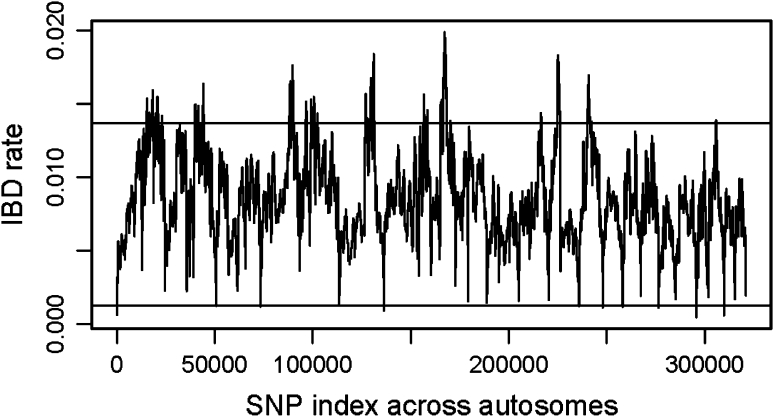
Rate of IBD segment detection across the autosomes in the NFBC data. Chromosomes are ordered left to right from 1 to 22. The IBD rate at a SNP is the number of pairs of individuals with an IBD segment covering the SNP, divided by the number of pairs of individuals in the data set. The *upper horizontal line* is the 95th percentile and is the cut-off for determining high IBD rate regions for the simulation study in Section 1.3 of the Appendix (Electronic supplementary material). The *lower horizontal line* is the 0.1th percentile and is the cut-off for weighting; regions with IBD rate below this threshold are excluded from the weighted estimates of relatedness. These low rates occur at positions with large gaps in the SNPs and/or poorer quality genotypes, such as telomeres and centromeres

One factor to consider in developing an IBD-segment-based estimator of relatedness is that the rate of IBD segment detection can vary from one point in the genome to another. These differing rates can be due to, for example, differing densities of genetic markers or differing strength of LD. If variants of large effect fall in regions of particularly high or low IBD segment detection, the estimates of heritability from IBD-segment-based methods can be biased [see Section 1.3 of the Appendix (Electonic supplementary material)]. At each point in the genome we calculated the proportion of pairs of individuals sharing an IBD segment covering the point (the IBD rate). Figure 1 shows that the IBD rate across the genome is quite variable. Therefore, in order to avoid the potential bias in heritability estimates, we down-weighted the IBD detected in regions with a high IBD rate and up-weighted the IBD detected in regions with a low IBD rate, so that each part of the genome contributes equally to relatedness estimates. We did, however, exclude regions with a very low IBD rate (IBD rate < *T*, where $T = 0.0013$, which is the 0.1th percentile of IBD rate in the NFBC data) because regions with extremely low rates of IBD detection would receive extremely high weights which would increase the variance of relatedness estimates. We also tried other values of the threshold *T*: the 2nd percentile ($T = 0.0031$) and the 5th percentile ($T = 0.0043$). There was very little difference in standard errors of the heritability estimates, so we chose to cover as much of the genome as reasonably possible using the 0.0013 threshold.

To calculate the weighted relatedness for a pair of individuals, we compute the following ratio:

2 $$ {\text{Relatedness}}_{\text{IBD}} (j,k) = \frac{{\sum\limits_{i} {1\left\{ {{\text{IR}}(i,i + 1) > T} \right\} \times {\text{IBD}}\left( {j,k,i,i + 1} \right) \times {\text{Length}}\left( {i,i + 1} \right)/{\text{IR}}\left( {i,i + 1} \right)} }}{{\sum\limits_{i} {2 \times 1\left\{ {{\text{IR}}(i,i + 1) > T} \right\} \times {\text{Length}}\left( {i,i + 1} \right)/{\text{IR}}\left( {i,i + 1} \right)} }} $$

where IR(*i*, *i*+1) is the IBD rate across the interval between SNPs *i* and *i*+1, $1\left\{ {{\text{IR}}\left( {i,i + 1} \right) > T} \right\}$ is 1 if the IBD rate is greater than the threshold *T* in this interval and 0 otherwise, ${\text{IBD}}\left( {j,k,i,i + 1} \right)$ is 1 if individuals *j* and *k* have a detected IBD segment that spans the interval between SNPs *i* and *i* + 1, and 0 otherwise. Length (*i*, *i* + 1) is the genetic length of the interval between SNPs *i* and *i* + 1, and the sums are over SNPs *i*, excluding the final SNP on each chromosome. The factor of two in the denominator is part of the definition of relatedness. Relatedness is twice the probability that a random allele taken from each of two individuals is IBD, or equivalently, ignoring the possibility of inbreeding, relatedness is half the probability that the two individuals are IBD through any pair of their alleles.

We also calculated an unweighted relatedness which was obtained by summing the genetic lengths of detected IBD segments for the pair and dividing by twice the genetic length of the genome (or equivalently, by replacing the IBD rate with a constant in the above equation). The weighted version was used except where otherwise noted.

Relatedness values for chromosome X are slightly different. We followed the approach of Yang et al. (2011) and used a full-dosage compensation model. For this model, IBD-segment-based relatedness values on chromosome X are the same as above for female–female pairs, multiplied by 2 for male–female pairs, and multiplied by 4 for male–male pairs. There is less IBD detected between male–male and male–female pairs because males have only one haplotype.

In the variance component approach, the diagonal values for the relatedness matrix (*A* in Eq. 1) are 1 + *f*, where *f* is the inbreeding coefficient for the individual. For the IBD-segment-based relatedness matrix, we estimate *f* by detecting segments of homozygosity by descent (HBD) using Beagle (Browning and Browning 2010) and, for each individual, dividing the sum of HBD lengths by the length of the genome.

### Principal components adjustment

We used GCTA version 0.93.9 (Yang et al. 2011) to calculate principal components to be used in adjustment for confounding of traits by population structure. First, we removed one individual from each pair of individuals identified as cousins or closer relationship from their IBD segments (see above). We calculated principal components and then identified outlying individuals for which the value in one of the first 20 eigenvectors was more than three times the interquartile range away from the median. 194 outliers were identified and removed. We then re-calculated principal components on the reduced set of individuals (without close relatives or outlying individuals). The first 20 principal components were used in subsequent estimation of heritability.

### Heritability analysis

We also used GCTA version 0.93.9 (Yang et al. 2011) to perform variance component-based estimation of heritability. Default settings were used except as otherwise noted. GCTA uses the SNP data to calculate a relatedness value for each pair of individuals based on allele-sharing (Yang et al. 2011). The estimated relatedness between individuals *j* and *k* is

3 $$ {\text{Relatedness}}_{\text{GCTA}} \left( {j,k} \right) = \frac{1}{N}\sum\limits_{i = 1}^{N} {\frac{{\left( {x_{ij} - 2p_{i} } \right)\left( {x_{ik} - 2p_{i} } \right)}}{{2p_{i} \left( {1 - p_{i} } \right)}}} $$

where *x*_*ij*_ is the number of copies of the reference allele at SNP *i* in individual *j*, *p*_*i*_ is the reference allele frequency for SNP *i* and *N* is the number of SNPs (Yang et al. 2011). Restricted maximum likelihood is used to fit the model of Eq. 1 to the data. *p* values for a test of whether the heritability is greater than zero are obtained via a likelihood ratio test in which the model with genetic effects is compared to a model without genetic effects.

The GCTA software documentation recommends not including the X chromosome in genome-wide analyses. We therefore performed autosome-wide analyses and separate X chromosome analyses.

We used the GCTA software to calculate the relatedness estimates given in Eq. 3 and to estimate heritability (GCTA method). We also calculated the weighted IBD-segment-based estimates of relatedness given in Eq. 2 and input these to GCTA for estimation of heritability (IBD-based variance component method). In these analyses, close relatives and principal components outliers were removed (see above) and we adjusted for 20 principal components.

Before applying the method of Zuk et al. (2012), we adjusted for covariates and for principal components eigenvectors by regressing the trait on the covariates and eigenvectors, and we standardized the residuals to have mean 0 and variance 1. In the method of Zuk et al. (2012) products of normalized trait values are regressed against relatedness values. The regression includes only pairs for which the relatedness value is not too far from the average relatedness value, to remove potential interactive effects. The estimated narrow-sense heritability equals the slope of the regression line at the average relatedness value multiplied by 1− the average kinship value, where the average kinship value is half the average relatedness value. In Section 1.2 of the Appendix (Electronic supplementary material), we present a proof of this equality for a purely additive genetic effect. Zuk et al. (2012) present an alternative proof of this equality for the general case where the genetic model includes interactions between variants and with environment. In all analyses, we excluded close relatives and individuals who were principal component outliers as for the GCTA analyses. We estimated the slope using pairs of individuals with relatedness values between 0 and twice the average relatedness value, as suggested by Zuk et al., except where otherwise noted. Heritability values must lie between zero and one, but the regression procedure can result in values outside this range. We set to zero any heritability estimates less than zero, and to one any heritability estimates greater than one. *p* values are obtained from testing whether the slope is significantly different from zero. Our analyses with the Zuk et al. method were performed in R (Ihaka and Gentleman 1996), using the lm() regression function to compute the slope, its standard error, and the *p* value. The estimated standard error of the slope and the *p* value from the lm() procedure assume independent observations which is not completely appropriate in this setting where observations are based on pairs of individuals, so that observations involving the same individual are not independent. A more rigorous approach would be to use a jackknife estimate of standard error (by repeating the analysis while leaving out entire individuals), but we do not use this approach here.

### Simulation study

We simulated phenotypes using the genotypes of the NFBC data. The simulation follows the additive model $y_{j} = \sum\nolimits_{i = 1}^{n} {\alpha_{i} \left( {x_{ij} - 2p_{i} } \right) + e_{j} }$, where *y*_*j*_ is the simulated trait value for individual *j*, *i* indexes the *n* causal SNPs, α_*i*_ is the effect of causal SNP *i*, *x*_*ij*_ is the number of copies of the reference allele at SNP *i* in individual *j*, *p*_*i*_ is the reference allele frequency for SNP *i*, and *e*_*j*_ is the environmental effect for individual *j*. We simulated a trait with *n* = 100 causal loci each contributing equally to the trait variance, and with total heritability 50 % for the trait. For each simulation replicate, 100 SNPs were randomly selected from the autosomal SNPs. We assigned 0.005 genetic variance to each of these 100 SNPs, which corresponds to $\alpha_{i} = \left( {400p_{i} \left( {1 - p_{i} } \right)} \right)^{ - 1/2}$. The effects of all the causal SNPs were added together, and a normally distributed environmental effect with mean zero and variance 0.5 was added. This results in total heritability of 50 %. A total of 400 replicate data sets were simulated. The 100 causal SNPs were chosen independently in each replicate.

## Results

### Heritability of metabolic traits in the North Finland Birth Cohort

We applied the Zuk et al. method (2012), the GCTA method (Yang et al. 2011), and the IBD-based variance component method to the NFBC data (5,402 individuals from Northern Finland). We estimated heritability for the metabolic traits measured in these data: BMI, HDL cholesterol, LDL cholesterol, systolic blood pressure, diastolic blood pressure, glucose, insulin, triglycerides and CRP. For each method, trait values were adjusted using 20 principal components to reduce confounding of environment with population structure.

Results for the autosome-wide analyses are shown in Table 1. Significant levels of heritability are seen for four of the traits: glucose (39 % heritability with IBD-based variance components), HDL cholesterol (46 %), LDL cholesterol (54 %), and BMI (16 % heritability with GCTA). For comparison the table also shows estimates of heritability based on twin studies for these traits. These twin-based results may be biased estimates of narrow-sense heritability due to a number of factors (Falconer and Mackay 1996) including the incorporation into the estimates of some interactive and dominance effects, and differences in environmental sharing between dizygotic and monozygotic twins. On the whole, these biases are expected to inflate the twin-based estimates, so it is not surprising that the twin-based estimates are larger than the narrow-sense heritability estimates we obtain from the data. Further factors could induce differences between the NFBC and twin-study estimates: the twin-based results are not from Northern Finland and heritability differs by population due to differing environmental factors and genetic variant frequencies; and the transformations of the traits to achieve approximate normality and the adjustment for covariates differ somewhat between the studies.

**Table 1:** Heritability estimates

Trait^a^	GCTA^b^	VC IBD^c^	Zuk et al.^d^	Twin studies^e^	Associated SNPs^f^
CRP	0.02 (0.06)^g^	0.08 (0.16)	0.00 (0.21)	0.56 (0.07) [W]	0.17
Glucose	0.18 (0.07)**^h^	0.39 (0.16)**	0.51 (0.22)*	0.67 (0.06) [S]	0.12
Insulin	0.07 (0.07)	0.04 (0.17)	0.03 (0.22)	0.49 (0.05) [S]	0.02
Triglycerides	0.08 (0.07)	0.00 (0.17)	0.00 (0.22)	0.65 (0.05) [W]	0.14
HDL	0.19 (0.07)**	0.46 (0.17)**	0.27 (0.22)	0.76 (0.06) [S]	0.21
LDL	0.29 (0.07)***	0.54 (0.17)***	0.10 (0.22)	0.78 (0.05) [S]	0.33
BMI	0.16 (0.07)**	0.00 (0.16)	0.00 (0.21)	0.80 (0.03) [W]	0.21
Diastolic	0.08 (0.07)	0.21 (0.16)	0.09 (0.21)	0.51 (0.06) [W]	0.00
Systolic	0.06 (0.06)	0.06 (0.16)	0.06 (0.21)	0.47 (0.06) [W]	0.00

^a^Traits have been transformed to adjust for covariates and achieve approximate normality, as described in “Subjects and methods”. Results from the twin studies have not necessarily had the same transformations/adjustments

^b^Estimates from the GCTA software using autosomal NFBC data

^c^Variance components approach using IBD-based estimates of relatedness from the autosomal NFBC data

^d^Regression approach of Zuk et al. using autosomal NFBC data

^e^Indicative estimates of heritability from twin studies taken from previous literature. Source is denoted by [W] (Wessel et al. 2007) or [S] (Souren et al. 2007). These estimates can differ from the true narrow-sense autosomal heritability of these traits in Northern Finland due to differences in environmental variances, differences in genetic make-up, incorporation of interaction effects or shared environment into family-based estimates, and contribution of the X chromosome

^f^Estimates of the proportion of trait variation in the NFBC data explained by SNPs significantly associated in the NFBC study or from previous studies are taken from Supplementary Table 1 of Sabatti et al. (2009). These estimates include effects from the X chromosome

^g^Estimates of heritability are given with standard errors in parentheses

^h^Statistical significance of estimates from this study are indicated by *single asterisk* (0.01 < *p* < 0.05), *double asterisks* (0.001 < *p* < 0.01), and *triple asterisks* (*p* *<* 0.001)

Estimates using variance component IBD-segment-based relatedness values are approximately 100 % higher than estimates using the GCTA method for a number of the traits including glucose, HDL cholesterol and LDL cholesterol, although 95 % confidence intervals for the estimates overlap. If reflecting true underlying differences, one plausible explanation for the difference is that rare variants contribute substantially to the heritability of these traits. Effects of rare variants may be included in the IBD-segment-based estimates of heritability whereas they are not well captured by the standard GCTA estimates.

**Fig. 2:**
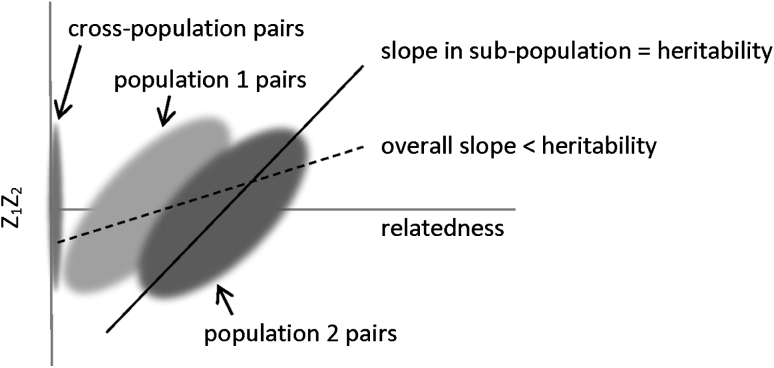
The potential effect of extreme population structure on regression-based heritability estimation. This figure illustrates an extreme scenario in which the heritability estimates of the method of Zuk et al. can be biased. In this scenario there are two sub-populations that have equal heritability. One sub-population has a higher rate of relatedness than the other. Pairs of individuals across populations have zero relatedness. Products of normalized trait values for pairs of individuals are shown on the *y*-axis, while relatedness values for the pairs are shown on the *x*-axis. It can be seen that, depending on the range of relatedness values used, the overall fitted slope will tend to be less than the heritability

The estimates from the method of Zuk et al. are significantly lower than those using IBD-segment based variance components for several of the traits (glucose is the one exception). Zuk et al. (2012) have shown that their method for estimating additive heritability is unbiased; however, the proof presented in Zuk et al. implicitly assumes that the population is homogenous. We hypothesized that the apparent bias observed when applying the Zuk et al. method to the NFBC is due to the effects of population structure. The use of principal components can largely correct for different mean trait values in different geographic regions due to environmental confounders. However, even without environmental confounding, regression-based methods for estimating heritability can be affected by population structure. Although the slope of the regression line in each sub-population may equal the heritability, if the sub-populations have different average levels of relatedness, the slope of a regression line applied to the full data set can be much less than the heritability, as illustrated in Fig. 2. In the simulation results presented later in this section we investigate this issue further, and demonstrate that the Zuk et al. estimator can be highly biased in the presence of population structure, even if there is no confounding with environmental factors. The NFBC data show strong population structure, with geographical regions largely separating on a multidimensional scaling (MDS) plot based on allele-sharing (Figure 1 of Sabatti et al. 2009).

We applied GCTA and IBD-segment-based variance components to the X chromosome. The only results that were significant at a 0.05-level were systolic blood pressure with a *p* value of 0.028 for the IBD-based analysis and 0.043 for the GCTA analysis, with estimated heritability 2.8 % (se 1.5 %) from the IBD-based analysis and 1.7 % (SE 1.1 %) from the GCTA analysis.

### Effects of population structure

To investigate further the performance of estimates of heritability in the NFBC data we performed simulations. We used the genotypes from the NFBC and created new traits based on additive genetic and random environmental effects, as described in “Subjects and methods”. As shown in Table 2, estimates from the Zuk et al. method are significantly lower than the true heritability on average. The GCTA estimates are slightly inflated, perhaps due to population structure biasing the estimation of relatedness (Manichaikul et al. 2010), while the IBD-based variance component estimates are approximately unbiased.

**Table 2:** Analysis of simulated polygenic phenotypes with NFBC genotypes

	Mean estimate^a^	SD^b^	Reported s.e.^c^
GCTA	0.517 (0.003)	0.061	0.059
VC IBD	0.500 (0.007)	0.146	0.151
Zuk et al.	0.296 (0.008)	0.164	0.195

Phenotypes are simulated to have 50 % heritability. Genotypic variance is equally distributed between 100 randomly selected, causal SNPs. Results are from 400 simulated replicates

^a^Mean estimated heritability with standard error in parentheses

^b^Standard deviation of estimates of heritability

^c^Average reported standard error of estimated heritability

Estimates from the Zuk et al. method differ depending on the range of relatedness values included in the regression. The results in Table 2 are for the standard range of 0 to twice average relatedness. Here average relatedness is 0.0033. We also calculated the estimates for different relatedness ranges, excluding relatives closer than cousins and individuals who are principal component outliers in all cases. When using all pairs remaining after these exclusions, the estimates become more biased, with mean 0.221 (SE 0.005). When using a more restricted range of relatedness values, estimates become less biased, however a high proportion of estimates are either 0 or 1, so that the estimates are not useful. For example, with relatedness restricted to 0.0016–0.0048 (0.5–1.5 times average relatedness), 30 % of estimates are 0 or 1, and the bias is not noticeably less (mean 0.336, SE 0.016), while with relatedness restricted to 0.0029–0.0035 (0.9–1.1 times average relatedness), 90 % of estimates are 0 or 1, and the mean estimated heritability is 0.460 (SE 0.024). Thus, if the sample size was much larger, one could use a very restricted range on relatedness and perhaps obtain an approximately unbiased estimate of heritability.

If population structure is the primary cause of the downward bias in the Zuk et al. estimate, we would expect to find less bias in a population with less structure. We simulated phenotypes using the WTCCC2 control data (5,200 individuals from the UK genotyped on a custom Illumina array with approximately one million SNPs), using the same phenotype simulation model as for the NFBC data. These data exhibit some population structure, but it is much less than that in Northern Finland. For example, the principal components values overlap significantly for different regions of the UK, whereas there is little overlap in the multi-dimensional scaling values for different regions in Northern Finland (Sabatti et al. 2009; The Wellcome Trust Case Control Consortium 2007). All three methods were approximately unbiased in these data (data not shown). However, due to the low level of IBD segments detected in these data, the standard errors of the IBD-segment-based estimates were extremely large, with most estimates taking values close to 0 or 1, so that these methods would not be useful in such data. In contrast, allele-sharing-based estimates have reasonable standard errors and have been applied successfully to data from the UK population (Lee et al. 2011).

## Discussion

We estimated the narrow-sense heritability of metabolic traits in the NFBC GWAS data. Several traits had high levels of estimated heritability, including LDL cholesterol, HDL cholesterol, glucose and BMI.

We contrasted IBD-segment-based estimates of relatedness with allele-sharing-based estimates of relatedness for the purpose of estimating heritability, and we compared variance-component-based estimates of heritability with regression-based estimates, using the NFBC data and simulated data. We found that IBD-segment-based estimates are significantly less precise (have higher standard errors) than allele-sharing-based estimates in the variance component approach. In the NFBC data, the reported standard errors of the heritability estimates were more than twice as large with IBD segments as with allele-sharing. We think that this is likely due to the effects of very distant relatedness. Very small segments of IBD (smaller than 1 cM) due to very distant relatedness (more than 50 generations) are usually not detectable and thus do not contribute to the IBD-segment-based estimates of relatedness; however, these segments will tend to contribute to allele-sharing-based estimates, albeit in a noisy fashion. In data from larger (more outbred) populations this effect is magnified because there is less recent relatedness. In the WTCCC2 data, the reported standard errors of variance component heritability estimates were approximately 14 times as large using IBD segments as using allele-sharing. This makes the use of IBD-segment-based methods for estimating heritability impractical in population data from a population with large effective size.

For several metabolic traits, we found that the IBD-segment-based estimates of heritability from variance components are substantially higher than allele-sharing-based estimates. While these differences were not statistically significant, this suggests that the IBD-segment-based estimates are incorporating genetic signals not captured by the allele-sharing-based estimates. A plausible explanation for this phenomenon is that the difference is due to the effects of rare variants which are not well captured by allele-sharing-based estimates. A substantial role of rare variants for metabolic traits is consistent with results from sequencing studies showing important contributions of rare variants to variation in several metabolic traits including HDL cholesterol (Cohen et al. 2004), LDL cholesterol (Cohen et al. 2006) and blood pressure (Ji et al. 2008).

IBD-based methods for estimating heritability depend on estimated IBD segments. A number of methods for IBD segment detection have been proposed (Purcell et al. 2007; Thomas et al. 2008; Kong et al. 2008; Gusev et al. 2009; Browning and Browning 2010, 2011a; Glazner and Thompson 2012), and typically are based on a length threshold or frequency threshold for matching haplotypes or genotypes. Here, we used a recently developed IBD segment detection method that will be included in the upcoming Beagle version 4 release.

Detected IBD segments are estimates, and are therefore subject to error. There will be some level of completely false-positive IBD segments. Using simulated and real data we have ascertained that this type of error is very rare with our method (unpublished data). Further, there will be some IBD segments that should fulfill our criteria (sufficiently long, or sufficiently rare) but that are not detected due to genotype errors or haplotype phasing errors, for example. Such errors add to noise but do not induce bias, provided that the rates of such errors are evenly distributed across the genome. However, we showed that the rate of detected IBD varies considerably over the genome in our results, and we showed [in Section 1.3 of the Appendix (Electronic supplementary material)] that reweighting in the relatedness estimation was necessary to avoid biases when variants of large effect fall in regions of high or low IBD detection. A third type of error is mis-determination of the IBD segment endpoints. Determination of endpoints of IBD segments is difficult, because a small number of genotyped markers may be consistent with IBD beyond the true end of the IBD segment, leading to false extension of the detected IBD segment. On the other hand, errors in genotypes or in haplotype phase may lead to premature truncation of the detected IBD segment. In simulated data, we have found that such effects balance each other out in the IBD detection method used here (unpublished data). Unbiased estimation of IBD segment length for detected IBD segments is important. If IBD segment length is overestimated, heritability will be underestimated, and vice versa.

We found that the regression-based method gave strongly downwardly biased estimates of heritability for simulated phenotypes in the NFBC data. Since this method implicitly assumes a homogenous population, we believe that the downward bias is due to the strong population structure in these data. In contrast, a variance component approach was fairly robust to population structure in the absence of environmental confounding in our analyses. It is possible that the variance-component-based heritability estimates in the real data could be upwardly biased due to environmental confounding with population structure, although previous studies suggest that most such environmental confounding effects are corrected by the use of principal components (Browning and Browning 2011b; Goddard et al. 2011).

The results reported here provide a salutary reminder that real data do not always conform to the assumptions underlying statistical methods. Methods which produced unbiased estimates in a homogenous population can produce biased estimates in a structured population. Incorporation of principal components into the analysis ameliorates bias due to environmental confounding but not other types of bias, such as bias due to population structure when estimating heritability when using the regression-based estimator of Zuk et al.

Finally, the differences between heritability estimates obtained in this study and heritability estimates from previous twin studies deserve further comment. For all nine metabolic traits examined, the twin-based estimates are higher than any of the GWAS-based estimates, although since the standard errors on the estimates are large, many of these differences are not individually statistically significant. The most extreme instance is BMI, with a twin-based estimate of 0.8, and GWAS-based estimates of up to 0.16. Taking estimates ±2 standard errors gives twin-based results implying heritability over 0.74 while the GWAS results imply heritability less than 0.42. Three factors potentially contribute to these differences. First, heritability can differ between populations. A twin-based study in Finland estimated heritability of BMI to be 0.69 in 30–39-year olds, which is little less than the 0.8 estimate but still significantly higher than the GWAS estimates (Schousboe et al. 2003); however, Northern Finland may differ from Finland as a whole. Second, twin-based estimates are known to partially incorporate non-additive genetic effects (interactive and dominance effects) and to be susceptible to other biases. Thus, our results could indicate a high contribution of interactive and dominance effects to the total genetic variance of these traits. Third, the GWAS heritability estimates may be downwardly biased. We showed that the regression-based (Zuk et al.) estimates are downwardly biased in the presence of population structure. The allele-sharing-based (GCTA) estimates primarily capture the effects of common variants, and thus are downwardly biased if rare variants contribute significantly to additive genetic variation. The IBD-segment-based variance component estimates should in principle incorporate effects of rare as well as common variation. However, the IBD-segment-based variance component estimates will miss some types of genetic variation. Variants in regions of the genome that are not well covered by SNPs on the GWAS array will not be incorporated in the estimates, although the proportion of the genome that is not well covered is small, so unlikely to greatly affect the estimates. Structural variants spanning multiple SNPs may significantly reduce the genotyping accuracy of the spanned SNPs, which will lead to loss of IBD segment detection and hence non-incorporation of the effects of these variants in the estimates. Thus, if the contribution of structural variants to the traits is large, the IBD-segment-based estimates will be downwardly biased.

## Electronic supplementary material

Below is the link to the electronic supplementary material.

Supplementary material 1 (PDF 144 kb)
