# Supplementary material for: Identity-by-descent-based heritability analysis in the Northern Finland Birth Cohort
Source: Hum Genet. 2012 Sep 29;132(2):129–38. doi: 10.1007/s00439-012-1230-y (PMC3543768; doi:10.1007/s00439-012-1230-y)
Supplement: Supplementary file 1 — Supplementary material 1 (PDF 144 kb) [file 439_2012_1230_MOESM1_ESM.pdf]

APPENDIX TO
IDENTITY-BY-DESCENT-BASED HERITABILITY ANALYSIS IN THE NORTHERN FINLAND BIRTH COHORT

SHARON R. BROWNING AND BRIAN L. BROWNING

1. Theoretical work relating to the method of Zuk et al. (2012)

1.1. **Haplotypes are conditionally independent in pairs of individuals for whom the genome-wide IBD sharing level is average.** In this section we prove that when one haplotype is selected from each of two individuals, the two haplotypes are independent if the genome-wide IBD sharing level for the two individuals is at the population-average level. This result assumes that the average rate of IBD detection is constant along the genome and that the population is unstructured. The result is used in Section 1.2 to prove the relationship between heritability and the slope of the regression line when the product of the trait values for pairs of samples is regressed on their genetic relatedness for an additive trait model (Zuk et al. provide a more general proof [1]). Let H_{ij} represent the binary allelic state of haplotype i at locus j. Similarly G_{ij} is the genotypic state (number of copies of the variant allele) of individual i at locus j, and Z_i is the individual's trait value. Let $\text{IBD}^G_{1,2}$ be the relatedness value for individuals 1 and 2 (twice the kinship coefficient; the kinship coefficient is the probability that a randomly selected allele from individual 1 and a randomly selected allele from individual 2 at the same locus are identical by descent), and let $\text{IBD}^H_{1,2}$ be the IBD proportion for haplotypes 1 and 2. Let $\text{IBD}^{Hj}_{1,2}$ be the IBD status at locus j for haplotypes 1 and 2. Define the average kinship coefficient,

$$k_0 = E(\text{IBD}^G_{1,2})/2 = E(\text{IBD}^H_{1,2}),$$

where the expectations are over pairs of individuals/haplotypes in the population, provided the IBD rate is constant over the genome and the population is homogeneous (unstructured). Note that,

$$P(\text{IBD}^{Hj}_{1,2} = 1|\text{IBD}^H_{1,2} = k_0) = k_0 = P(\text{IBD}^{Hj}_{1,2} = 1).$$

Similarly, if haplotypes 1 and 2 are randomly chosen from two individuals with relatedness $\text{IBD}^{G}_{1,2}$,

$$P(\text{IBD}^{Hj}_{1,2} = 1|\text{IBD}^{G}_{1,2} = 2k_0) = P(\text{IBD}^{Hj}_{1,2} = 1|\text{IBD}^{H}_{1,2} = k_0) = k_0.$$

We will show that

$$P(H_{2j} = h_{2j}|H_{1j} = h_{1j}, \text{IBD}^{H}_{1,2} = k_0) = P(H_{2j} = h_{2j}|\text{IBD}^{H}_{1,2} = k_0),$$

and hence that

$$P(H_{1j} = h_{1j}, H_{2j} = h_{2j}|\text{IBD}^{G}_{1,2} = 2k_0) = P(H_{1j} = h_{1j})P(H_{2j} = h_{2j}).$$

Overall in the population, haplotypes are assumed to be independent. Thus,

$$P(H_{2j} = h_{2j}|H_{1j} = h_{1j}) = P(H_{2j} = h_{2j}).$$

We assume that the probability of IBD does not depend on the allele carried by one of the individuals:

$$P(\text{IBD}^{Hj}_{1,2} = 1|H_{1j} = h_{1j}) = P(\text{IBD}^{Hj}_{1,2} = 1)$$

which holds provided there is no population structure. We now have that

$$\begin{aligned}
&P(H_{2j} = h_{2j}|H_{1j} = h_{1j}, \text{IBD}^{H}_{1,2} = k_0)\\
&\quad = P(H_{2j} = h_{2j}|H_{1j} = h_{1j}, \text{IBD}^{Hj}_{1,2} = 1)P(\text{IBD}^{Hj}_{1,2} = 1|\text{IBD}^{H}_{1,2} = k_0)\\
&\qquad\qquad + P(H_{2j} = h_{2j}|H_{1j} = h_{1j}, \text{IBD}^{Hj}_{1,2} = 0)P(\text{IBD}^{Hj}_{1,2} = 0|\text{IBD}^{H}_{1,2} = k_0)\\
&\quad = P(H_{2j} = h_{2j}|H_{1j} = h_{1j}, \text{IBD}^{Hj}_{1,2} = 1)P(\text{IBD}^{Hj}_{1,2} = 1)\\
&\qquad\qquad + P(H_{2j} = h_{2j}|H_{1j} = h_{1j}, \text{IBD}^{Hj}_{1,2} = 0)P(\text{IBD}^{Hj}_{1,2} = 0)\\
&\quad = P(H_{2j} = h_{2j}|H_{1j} = h_{1j}, \text{IBD}^{Hj}_{1,2} = 1)P(\text{IBD}^{Hj}_{1,2} = 1|H_{1j} = h_{1j})\\
&\qquad\qquad + P(H_{2j} = h_{2j}|H_{1j} = h_{1j}, \text{IBD}^{Hj}_{1,2} = 0)P(\text{IBD}^{Hj}_{1,2} = 0|H_{1j} = h_{1j})\\
&\quad = P(H_{2j} = h_{2j}, \text{IBD}^{Hj}_{1,2} = 1|H_{1j} = h_{1j}) + P(H_{2j} = h_{2j}, \text{IBD}^{Hj}_{1,2} = 0|H_{1j} = h_{1j})\\
&\quad = P(H_{2j} = h_{2j}|H_{1j} = h_{1j})\\
&\quad = P(H_{2j} = h_{2j})\\
&\quad = P(H_{2j} = h_{2j}|\text{IBD}^{H}_{1,2} = k_0).
\end{aligned}$$

That is, H_{1j} and H_{2j} are conditionally independent when the kinship coefficient of the haplotypes is at the population average level. In the above proof, we can replace $\text{P}(\text{IBD}^{Hj}_{1,2} = x|\text{IBD}^{H}_{1,2} = k_0)$ with $\text{P}(\text{IBD}^{Hj}_{1,2} = x|\text{IBD}^{G}_{1,2} = 2k_0)$, where $\text{IBD}^{G}_{1,2}$ is the relatedness for the pair of individuals from whom the two haplotypes are

randomly chosen, since these two probabilities are equal. Hence the haplotypes are conditionally independent when the relatedness of the individuals from whom the haplotypes are randomly selected is at the population average level:

$$P(H_{2j}=h_{2j},H_{1j}=h_{1j}|\text{IBD}^G_{1,2}=2k_0)=P(H_{1j}=h_{1j})P(H_{2j}=h_{2j}).$$

1.2. **Regression line slope and heritability for an additive trait.** Let Z_i be the normalized trait value (mean zero, variance one) for individual i. Suppose the genetic contribution to Z_i is purely additive. We can write

$$Z_i=\alpha+\sum\beta_j(H^m_{ij}+H^p_{ij})+\varepsilon_i,$$

where H^m_{ij} is the maternally inherited allele of individual i at locus j, H^p_{ij} is the paternally inherited allele, β_j is the effect of locus j, and ε_i is the environmental effect for individual i, which has mean zero, variance σ^2_ε, and is independent of all genotypes and all other individuals' environmental effects. Let p_j be the allele frequency at locus j. In order for Z_i to have mean zero and variance one, we require that

$$\alpha=-E(\sum\beta_j(H^m_{ij}+H^p_{ij}))=-2\sum\beta_jp_j$$

and

$$2\sum\beta_j^2p_j(1-p_j)=1-\sigma^2_\varepsilon.$$

Note that the heritability h^2 of the trait is the variance of

$$\sum\beta_j(H^m_{ij}+H^p_{ij})$$

which is

$$2\sum\beta_j^2p_j(1-p_j).$$

Using independence and zero mean of the environmental effect and

$$\alpha=-E\left(\sum\beta_j(H^m_{ij}+H^p_{ij})\right),$$

we obtain

(1)

$$\begin{aligned}
&E(Z_1Z_2|\text{IBD}^G_{1,2}=r)\\
&\quad=E\left(\left(\alpha+\sum\beta_j(H^m_{1j}+H^p_{1j})+\varepsilon_1\right)\left(\alpha+\sum\beta_j(H^m_{2j}+H^p_{2j})+\varepsilon_2\right)\middle|\text{IBD}^G_{1,2}=r\right)\\
&\quad=-\alpha^2+\sum\beta_j^2E\left(H^m_{1j}H^m_{2j}+H^m_{1j}H^p_{2j}+H^p_{1j}H^m_{2j}+H^p_{1j}H^p_{2j}\,\middle|\text{IBD}^G_{1,2}=r\right).
\end{aligned}$$

Now H_{1j} and H_{2j} are independent when $\text{IBD}^H_{1,2} = k_0$, as shown in Section 1.1. Thus

$$E(H_{1j}H_{2j}|\text{IBD}^H_{1,2} = k_0) = E(H_{1j})E(H_{2j}) = p_j^2.$$

Also,

$$E(H_{1j}H_{2j}|\text{IBD}^{Hj}_{1,2} = 1) = p_j.$$

Now

$$\begin{aligned}
p_j^2 &= E(H_{1j}H_{2j}|\text{IBD}^H_{1,2} = k_0) \\
&= E(H_{1j}H_{2j}|\text{IBD}^{Hj}_{1,2} = 1)P(\text{IBD}^{Hj}_{1,2} = 1|\text{IBD}^H_{1,2} = k_0) \\
&\qquad + E(H_{1j}H_{2j}|\text{IBD}^{Hj}_{1,2} = 0)P(\text{IBD}^{Hj}_{1,2} = 0|\text{IBD}^H_{1,2} = k_0) \\
&= p_j k_0 + E(H_{1j}H_{2j}|\text{IBD}^{Hj}_{1,2} = 0)(1 - k_0).
\end{aligned}$$

Solving the above equation, we find that

$$E(H_{1j}H_{2j}|\text{IBD}^{Hj}_{1,2} = 0) = (p_j^2 - p_j k_0)/(1 - k_0).$$

Then

$$\begin{aligned}
E\left(H_{1j}H_{2j}\,|\text{IBD}^G_{1,2} = r\right) &= E\left(H_{1j}H_{2j}\,|\text{IBD}^H_{1,2} = r/2\right) \\
&= E\left(H_{1j}H_{2j}\,\middle|\text{IBD}^{Hj}_{1,2} = 1\right)P(\text{IBD}^{Hj}_{1,2} = 1\,|\text{IBD}^H_{1,2} = r/2) \\
&\qquad + E\left(H_{1j}H_{2j}\,\middle|\text{IBD}^{Hj}_{1,2} = 0\right)P(\text{IBD}^{Hj}_{1,2} = 0\,|\text{IBD}^H_{1,2} = r/2) \\
&= p_j r/2 + (p_j^2 - p_j k_0)(1 - r/2)/(1 - k_0) \\
&= r p_j(1 - p_j)/(2(1 - k_0)) + p_j(p_j - k_0)/(1 - k_0).
\end{aligned}$$

Thus, substituting into Equation 1,

$$\begin{aligned}
&E(Z_1Z_2|\text{IBD}^G_{1,2} = r) \\
&\quad = -\alpha^2 + \sum 4\beta_j^2 p_j(p_j - k_0)/(1 - k_0) + \sum 2\beta_j^2 p_j(1 - p_j)r/(1 - k_0) \\
&\quad = -\alpha^2 + \sum 4\beta_j^2 p_j(p_j - k_0)/(1 - k_0) + h^2 r/(1 - k_0).
\end{aligned}$$

Thus the regression of Z_1Z_2 on relatedness, r, is linear with slope $h^2/(1 - k_0)$.

1.3. **What happens when the IBD rate varies by locus?** Suppose the rate of IBD varies by locus, with

$$\text{P}(\text{IBD}^{Hj}_{1,2} = 1|\text{IBD}^H_{1,2} = k) = \gamma_j k,$$

and

$$\sum_{j=1}^{n} \gamma_j/n = 1,$$

where n is the number of SNPs. Then, a similar calculation as that shown above gives

$$E\left(H_{1j}H_{2j}\left|\text{IBD}^G_{1,2}=r\right.\right)=\gamma_j r p_j(1-p_j)/2(1-\gamma_j k_0)+C$$

where C is a constant. Thus, for an additive trait in a homogeneous population,

$$E(Z_1Z_2|\text{IBD}^G_{1,2}=r)=C+\sum 2\beta_j^2 p_j(1-p_j)\gamma_j r/(1-\gamma_j k_0).$$

If some loci have a large contribution to heritability, the estimate of heritability will be biased up or down depending on whether those loci are in regions with high or low IBD rate.

We hypothesized that this effect would also extend to the IBD-based variance-components method. To investigate the magnitude of the effect, we simulated a trait for the Northern Finland Birth Cohort (NFBC) data in a similar manner to the simulation in the main text, but with only one causal locus. The locus was selected from those loci with estimated IBD proportion in the top 5% (see Fig. 1 in main text). For each simulation replicate, one such SNP with minor allele frequency greater than 0.05 was randomly selected, and given an additive effect with effect $\beta=1/\sqrt{4p(1-p)}$, where p is the allele frequency for the SNP. The mean was subtracted and a normally distributed environmental effect with mean zero and variance 0.5 was added. This results in total heritability of 50%. A total of 100 replicate data sets were simulated. When using unweighted relatedness estimates (see Methods in main text) in the IBD-based variance-components method, the mean estimated heritability was 0.675 (s.e. 0.016), which is significantly inflated. When using weighted relatedness estimates, the mean estimated heritability was 0.456 (s.e. 0.015). This is slightly deflated, perhaps because the polygenic assumption of the variance components model is violated here.

2. Parameters used in IBD analysis of the Northern Finland Birth Cohort data

We used a new (not yet published) version of Beagle to detect IBD in the NFBC data. Briefly, the method phases the data, and looks for matching haplotypes longer than a given length threshold. Up to this point it is very similar to Germline [2]. It then runs a likelihood ratio test for IBD versus non-IBD using the Beagle model. As a likelihood ratio test threshold we used 1000: only segments that are at least 1000 times more likely under the IBD model than under the non-IBD model

are reported to be IBD. We use a scale factor of 12 when building the Beagle model for the likelihood ratio test. This is much larger than scale factors that we have used in previous work (1 or 2) [3]. We have found that it is desirable to increase the scale factor with increasing sample size and also with increasing levels of relatedness in the sample. In order to choose the appropriate scale factor, we masked every 10th marker, inferred IBD, and then observed whether the masked markers were consistent with the detected IBD (i.e. not homozygous discordant). As the scale factor increases, the error rate increases, however we found that as we increased the scale factor the error rates remained reasonable, and the amount of IBD found did not increase much further beyond a scale factor of 12. We used a length threshold of 2 cM for the first part of the IBD detection, because in analysis of chromosome 20 data, all segments passing the likelihood ratio test had length greater than this threshold, and applying a stringent threshold at this stage reduces downstream computation.

3. Further heritability estimates from the Northern Finland Birth Cohort data

In this section we provide estimates of heritability obtained from variant parameter settings for the methods used in the main text. Results from Table 1 in the main text are included for comparison. Except as otherwise noted, all analyses are as described in Methods in the main text. For example, relatives closer than cousins and principal component (PC) outliers are excluded, and analyses adjust for sex and oral contraceptive use.

The GCTA software [4] provides an extrapolation that increase the heritability estimate to the extent that would be expected if all variants were genotyped and all causal SNPs followed the same minor allele frequency distribution as the genotyped SNPs in the original data set. These estimates are given in the third column of Table A1 below, and can be compared to the original results in the second column of this table (also in Table 1 in the main text). It can be seen that the differences are small: at most 0.03. This indicates that due to high levels of linkage disequilibrium, common variants are already well tagged in these data, and a higher density of common SNPs would not reveal much additional heritability.

Zuk et al. [1] recommend applying the regression for their heritability estimate only to pairs of individuals with close to average relatedness. As in their example,

we restricted this analysis to pairs with relatedness less than twice the average. They caution that including close relatives can inflate estimates of heritability due to the incorporation of non-additive effects. In the sixth column of Table A1 we show the results of analysis when all pairs are used, except that cousins and closer relatives as well as PC outliers were excluded. The original restricted analysis results are shown in the fifth column for comparison. Including these additional pairs reduces the standard errors of the estimates. Contrary to expectations, some of the estimates decrease substantially with the reduction in the restriction on relatedness. This may be a reflection of the population structure effects. In the simulated trait data, we found that restricting the relatedness range has the effect of reducing the population-structure induced downward bias, although standard errors also become higher. It is not possible to increase the restriction on relatedness range beyond the original restriction, as the standard errors become too high for the results to be useful.

Several of the traits were log-transformed in the original analyses presented in Table 1 of the main text. In this table we include results for unlogged traits for comparison. These analyses were still adjusted for age and contraceptive use, and were windsorized as for the other traits. Except for CRP with the VC IBD method, the logged and unlogged analyses yielded almost identical results.

Table A1. Extended heritability analyses of the NFBC data (estimates with standard errors)

Trait	GCTA	GCTA extrapolated	VC IBD	Zuk et al. restricted relatedness	Zuk et al. unrestricted relatedness
CRP	0.03 (0.06)	0.03 (0.07)	0.25 (0.16)	0.07 (0.21)	0.07 (0.12)
log(CRP)	0.02 (0.06)	0.02 (0.07)	0.08 (0.16)	0.00 (0.21)	0.00 (0.12)
Glucose	0.18 (0.07)**	0.20 (0.07)**	0.40 (0.16)**	0.55 (0.22)*	0.18 (0.13)
log(Glucose)	0.18 (0.07)**	0.20 (0.07)**	0.39 (0.16)**	0.51 (0.22)*	0.17 (0.13)
Insulin	0.05 (0.07)	0.05 (0.07)	0.04 (0.16)	0.03 (0.22)	0.00 (0.13)
log(Insulin)	0.07 (0.07)	0.07 (0.07)	0.00 (0.17)	0.00 (0.22)	0.00 (0.13)
Triglycerides	0.08 (0.07)	0.09 (0.08)	0.00 (0.17)	0.00 (0.22)	0.00 (0.13)
log(Trigly.)	0.08 (0.07)	0.08 (0.08)	0.00 (0.17)	0.00 (0.22)	0.00 (0.13)
HDL	0.19 (0.07)**	0.21 (0.07)**	0.46 (0.17)**	0.27 (0.22)	0.17 (0.13)
LDL	0.29 (0.07)***	0.32 (0.08)***	0.54 (0.17)***	0.10 (0.22)	0.22 (0.13)
BMI	0.16 (0.07)**	0.18 (0.07)**	0.03 (0.16)	0.00 (0.21)	0.00 (0.12)
log(BMI)	0.16 (0.07)**	0.18 (0.07)**	0.00 (0.16)	0.00 (0.21)	0.00 (0.12)
Diastolic	0.08 (0.07)	0.08 (0.07)	0.21 (0.16)	0.09 (0.21)	0.04 (0.12)
Systolic	0.06 (0.06)	0.06 (0.07)	0.06 (0.16)	0.06 (0.21)	0.00 (0.12)

Statistical significance of estimates is indicated by * $0.01<p<0.05$; ** $0.001<p<0.01$; *** $p<0.001$.

Literature cited

[1] Zuk O, Hechter E, Sunyaev SR, Lander ES (2012) The mystery of missing heritability: Genetic interactions create phantom heritability. Proc Natl Acad Sci U S A.

[2] Gusev A, Lowe JK, Stoffel M, Daly MJ, Altshuler D, et al. (2009) Whole population, genome-wide mapping of hidden relatedness. Genome Res 19: 318-326.

[3] Browning BL, Browning SR (2011) A fast, powerful method for detecting identity by descent. Am J Hum Genet 88: 173-182.

[4] Yang J, Lee SH, Goddard ME, Visscher PM (2011) GCTA: a tool for genome-wide complex trait analysis. American Journal of Human Genetics 88: 76-82.
